# Time to Relapse and Its Predictors among Children with Nephrotic Syndrome in Comprehensive Specialized Hospitals, Tigray, Ethiopia, 2019

**DOI:** 10.1155/2020/8818953

**Published:** 2020-11-22

**Authors:** Miliete Gebrehiwot, Mekuria Kassa, Haftom Gebrehiwot, Migbar Sibhat

**Affiliations:** ^1^Department of Nursing, College of Health Sciences and Medicine, Adigrat University, Adigrat, Ethiopia; ^2^School of Nursing, College of Health Sciences, Mekelle University, Mekelle, Ethiopia; ^3^Department of Nursing, College of Health Sciences and Medicine, Dilla University, Dilla, Ethiopia

## Abstract

**Background:**

Relapse in children with nephrotic syndrome leads to a variety of complications due to prolonged treatment and potential dependency on steroids. However, there is no study conducted to determine the incidence and predictive factors of relapse for nephrotic syndrome in Ethiopia, especially in children. Thus, this study aimed to assess the incidence of relapse and its predictors among children with nephrotic syndrome in Ethiopia.

**Methods:**

A retrospective study was conducted by reviewing all charts of children with an initial diagnosis of the nephrotic syndrome in tertiary hospitals from 2011 to 2018. Charts of children with a diagnosis of steroid-resistant cases were excluded. The extraction tool was used for data collection, Epi-data manager V-4.4.2 for data entry, and Stata V-14 for cleaning and analysis. Kaplan-Meier curve, log-rank test, life table, and crude hazard ratios were used to describe the data and adjusted hazard ratios with 95% CI and *P* value for analysis. Median relapse time, incidence rate of relapse, and cumulative relapse probabilities at a certain time interval were computed. Bivariable and multivariate analyses were performed using the Cox proportional hazard regression to identify the factors associated with relapse. Any variable at *P* < 0.25 in the bivariable analysis was transferred to multivariate analysis. Then, the adjusted hazard ratio with 95% CI and *P* ≤ 0.05 was used to report the association and to test the statistical significance, respectively. Finally, texts, tables, and graphs were used to present the results. *Results and Conclusion*. Majority, 64.5% (40/66), of relapses were recorded in the first 12 months of follow-up. The incidence rate of relapse was 42.6 per 1000 child-month-observations with an overall 1454 child-month-observations and the median relapse time of 16 months. Having undernutrition [AHR = 3.44; 95% CI 1.78-6.65], elevated triglyceride [AHR = 3.37; 95% CI 1.04-10.90], decreased serum albumin level [AHR = 3.51; 95% CI 1.81-6.80], and rural residence [AHR = 4.00; 95% CI 1.49-10.76] increased the hazard of relapse. *Conclusion and Recommendation*. Relapse was higher in the first year of the follow-up period. Undernutrition, hypoalbuminemia, hypertriglyceridemia, and being from rural areas were independent predictors of relapse. A focused evaluation of those predictors during the initial diagnosis of the disease is compulsory.

## 1. Background

Nephrotic syndrome is a chronic relapsing renal disease in children with a higher incidence in low-income countries. It is one of the common causes of pediatric mortality and morbidity in such countries [1–4]. It is characterized by heavy proteinuria (40 mg/m2/hr in children), hypoalbuminemia (<2.5 g/dl), peripheral edema, and hyperlipidemia [5, 6]. Nephrotic syndrome (NS) affects children of any age and is most commonly seen among school-age children and adolescents [7]; although, the risk of relapse decreases in adolescents [8].

More than half of children with nephrotic syndrome suffer one or more relapses and most of them occur within the first six months [9, 10]. Around 80% of children with steroid-sensitive nephrotic syndrome experience relapse in developed countries such as Italy and Australia [11, 12]. It is also high in middle-income and sub-Saharan African countries [13–15]. Relapse in children with nephrotic syndrome leads to different complications such as hypertension, cataracts, osteoporosis, overweight, growth retardation [16], infertility [17], and hearing impairment [18]. However, these complications may occur either directly due to the disease course or indirectly from the steroid and immunosuppressive therapies. It is also a psychological burden for the child as well as an economic, social, and emotional burden for the families due to repeated hospital visits, the need for a prolonged hospital stay, service payments, and generally its effect on the quality of life of the family [19–21].

Globally, different trials were undergone to reduce the risk of relapse in children with nephrotic syndromes, such as zinc supplementation [22] and daily administration of prednisolone therapy [6] during the episode of upper respiratory infection. However, the reduction and control of relapse in children with the nephrotic syndrome remain a global challenge [13].

In Ethiopia, nephrotic syndrome is the second most common cause of pediatric admissions among those diagnosed with renal disease [23]. Nevertheless, there is no intervention being provided in Ethiopia except for the daily administration of prednisolone. Furthermore, there is no trial or study conducted regarding this issue in the Tigray region and even throughout the country except for a single prevalence study done at Tikur Anbesa comprehensive specialized hospital. On the other hand, because of the relapsing nature of the disease, many children with nephrotic syndrome are visiting health institutions repeatedly and are exposed to unwanted economic and social problems. Thereupon, we need to research to determine the incidence of relapse and to identify factors that provoke relapse in children with nephrotic syndrome.

## 2. Methods

A retrospective follow-up study was conducted at comprehensive specialized hospitals of the Tigray region, Ethiopia, by reviewing eight years of follow-up data on charts of children with an initial diagnosis of the nephrotic syndrome from 2011 to 2018. According to the 2018 report, the total population of the Tigray region was 5,377,144, of which 2,349,812 were under 15 years of age [24]. The region has two comprehensive specialized hospitals: Aksum comprehensive specialized hospital and Ayder comprehensive specialized hospital. Ayder serves people who come from Tigray, Afar, southeastern parts of the Amhara region, and the Eritrean refugees, whereas Aksum referral hospital serves patients who come from central, western, and northwest parts of Tigray. There are no specific institutional guidelines developed to manage the nephrotic syndrome. Health professionals provide diagnostic and therapeutic services based on international standards and recommendations. After the confirmation of nephrotic syndrome, children were followed-up and monitored at renal clinics. The dosage and duration of therapy as well as the frequency of visits varied from patient to patient. Two hundred fifty (250) children had nephrotic syndrome in both hospitals during the follow-up period (2011–2018). The study was conducted from December 2018 to July 2019.

### 2.1. Study Population and Recruitment Methods

**Figure 1:**
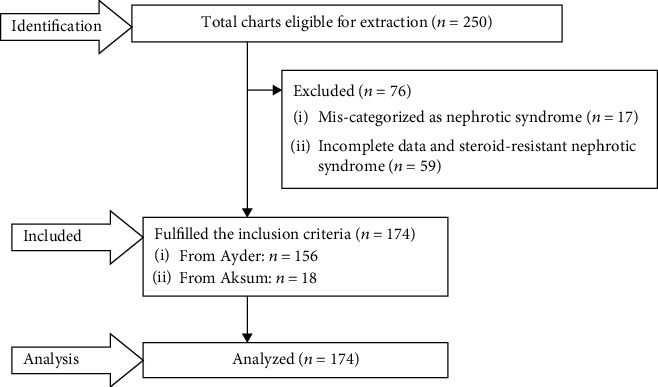
A flow chart of recruitment methods.

The study population included all children below 18 years of age who were newly diagnosed with idiopathic nephrotic syndrome from the beginning of 2011 to the end of 2018 in comprehensive specialized hospitals of the Tigray region. The lists of charts were obtained from the health service management information system database. Only the first relapse after the initial diagnosis of nephrotic syndrome was recorded as an event, and data were not collected after the occurrence of the first relapse; thus, children with multiple relapses were not considered more than once. Charts of children with missing data (e.g., proteinuria) and those diagnosed with steroid-resistant nephrotic syndrome (SRNS) were excluded. This was because there is no precise treatment guideline for optimal therapy; thus, it is difficult to predict outcomes. The details of steps and procedure of study participants were described in the figure below (Figure 1).

### 2.2. Operational Definitions and Measurements

Events were children with nephrotic syndrome who experienced the first relapse after the achievement of remission. Remission was having <1+ proteinuria for 3 consecutive days after the commencement of treatment [6]. Relapse was the recurrence of ≥3+ proteinuria for three consecutive days after having been in remission and declared when the patients' records indicated relapse and/or when the diagnostic criteria were fulfilled irrespective of therapeutic modifications [6]. Steroid sensitivity was defined as the achievement of complete remission within four weeks of steroid therapy after initial presentation. Steroid resistance was the absence of remission despite therapy with daily corticosteroid therapy [25, 26]. Censored were those cases that had never experienced a relapse during follow-up including dropouts, transferred outs, died, exceed 18 years of age during follow-up, and those who did not achieve remission yet. Survival time (time to relapse or censor) was measured from the initial diagnosis of the nephrotic syndrome to the occurrence of the first relapse. The time from initiation of follow-up to the occurrence of relapse or censored cases was measured in months. For children above six years, acute malnutrition (wasting) was classified into severe acute malnutrition (SAM) and moderate acute malnutrition (MAM) according to the degree of wasting and the presence of edema. SAM was diagnosed if *z*-score of weight-for-height (WFH) <-3 or midupper arm circumference (MUAC) <110 mm, or there is bilateral pitting edema and MAM if WFH between -3 and -2 *z*-score without pitting edema [27]. BMI for age (BAZ) below -2 *z*-score was considered to diagnose wasting for children under six years. Underweight was considered when the child had weight-for-age (WFA) less than -2 *z*-score whereas stunted if the z-score of height-for-age (HFA) of a child below -2 [28]. Children were declared as having hypertension if stated under their medical records. The presence or absence of infection was also determined based on the diagnosis noted on the patients' charts.

### 2.3. Data Collection Instruments and Procedure

The information available in the patient charts was checked first and an appropriate data extraction checklist was prepared in English. The checklist was adapted from the renal clinic follow-up registration book and by reviewing different related literature and articles. The extraction tool comprised of sociodemographic characteristics (such as age at onset of the disease, sex, residence, and family history of renal disease), disease-related characteristics (such as regimen of initial steroid therapy, numbers of days to achieve remission, history of atopic disease, infection, HTN, duration of treatment, and malnutrition), and biochemical related variables (serum albumin, total protein, hematuria, and hypertriglyceridemia). The lists of children with nephrotic children were obtained from the follow-up registration books of the renal clinic and pediatric wards. Three data collectors (bachelor nurses working out of the selected hospitals) and one supervisor (masters student) were hired, and the data collection was accomplished within three weeks from April 7 to 26/2019.

### 2.4. Data Processing, Analysis, Interpretation, and Presentation

As soon as the data collection was completed, the collected data were checked thoroughly by observation for any unfilled or inappropriate responses. Next, the data were entered into Epi-data manager version 4.4.2.1 and exported to STATA version 14 for cleaning, edition, coding, and analysis. The nature of data such as normality and the presence of outliers as well as the levels of missing values were determined before description and analysis of data. Then, the data were described using relative frequency, percent, mean with standard deviation, and median based on its applicability. Life table was used to estimate the cumulative probabilities of relapse at different time intervals. Kaplan-Meier's relapse curve was considered to estimate median relapse time during the follow-up period and log-rank tests to compare survival curves for the presence of difference in the incidence of relapse among the groups.

The bivariable analysis was carried out to identify possible associations between relapse and each covariate. Those variables having *P* ≤ 0.25 were transferred to multivariate analysis to identify independent predictors of relapse.

Multicollinearity between independent variables was checked using the variance inflation factor (meanvif = 1.24), and all were within the acceptable range. The proportional hazard assumptions were also tested using a global test with a value of *P* > *χ*^2^ = 0.96 which was highly insignificant. Finally, the Cox regression model for its fitness to the data was checked using the Cox-Snell residuals, and the hazard function follows the 45-degree line very closely. Generally, we could conclude that the final model fits the data successfully. In the multivariate analysis, any statistical test was considered significant at *P* ≤ 0.05. Then, the association between relapse and independent variables was declared by using an adjusted hazard ratio with 95% CI. Finally, texts, tables, and graphs were used to present the results.

### 2.5. Data Quality Assurance

Before starting the actual data collection, a pretest was conducted on thirteen of randomly selected charts at Mekelle general hospital, which was not included in the actual study. One day training was given for data collectors and a supervisor focusing on what information to be collected and the way they were going to collect relevant information. The Appraisal of the collected data was done at the end of each day by the principal investigator and supervisor for completeness. Charts with incomplete data during data collection were excluded.

### 2.6. Ethical Consideration

Ethical approval was received from the institutional review board (IRB) of Mekelle University, college of health sciences. Then, an official letter of cooperation was written to Ayder and Aksum comprehensive specialized hospitals from the school of nursing, and permission for data collection was obtained from selected hospitals on behalf of patients and renal clinics, and pediatric wards of each hospital since the study was conducted through a review of medical records. Finally, data were collected from the charts of children with nephrotic syndrome. For certainty of anonymity and confidentiality, data were coded and reported as combined.

## 3. Results

From the beginning of 2011 to the end of 2018, there were 174 children newly diagnosed with an idiopathic nephrotic syndrome that fulfilled the inclusion criteria and were included in the study for analysis. The study participants had a minimum follow-up of 1 month and a maximum of 67 months.

### 3.1. Sociodemographic Characteristics

The findings of this study showed that ninety-eight (56.3%) participants were male and 76 (43.7%) were female. Among male participants (*n* = 98), 59 (60.2%) children experienced relapse. Fifty-nine (33.9%) children were diagnosed with nephrotic syndrome after the age of nine with a median age of seven years. Besides, fifty-six (32.2%) children were diagnosed with nephrotic syndrome at early school age (7-9 years), of which 42.9% (24/56) experienced relapse, whereas 39.1% (9/23) of toddlers (1-3 years) with nephrotic syndrome developed relapse. The result also showed that among the total 174 participants, 123 (70.7%) children came from rural areas. Of those who came from rural areas (*n* = 123), fifty-five (44.7%) study subjects developed relapse. None of the study participants had a known family history of renal diseases (Table 1).

### 3.2. Baseline Clinical (Diseases Related) Characteristics

Among ninety (51.7%) children who had hypertension initially at the diagnosis of nephrotic syndrome, 35.6% (32/90) developed relapse. Of the 102 (58.6%) children who had an underline infection, 58 (57%) children experienced a relapse. Meanwhile, among those children who received a treatment course of less than 8 weeks (*n* = 39), 19 (41%) experienced relapse, and 69% (20/29) children among those having a history of allergy had developed relapse. All of the study participants (*n* = 174) had taken prednisolone steroid therapy for the initial time and the median duration of steroid treatment was 8 weeks. Thirty-seven (49.3%) children who experienced relapse achieved remission after 2 weeks from the commencement of therapy and 38 (50.7%) from the censored group achieved remission after 14 days of treatment initiation (Table 2).

### 3.3. Biochemical-Related Characteristics of the Study Participants

One hundred fifty-eight (90.8%) participants had a serum protein level of 3-6 g/dl at the initial diagnosis. Among those children who had baseline serum protein levels of 3-6 g/dl, 34.8% (55/158) developed relapse. On the other hand, of those children having a serum protein level below 3 g/dl, 62.5% (5/8) reported as relapsed. Moreover, 43 (24.7%) children had serum albumin level of ≤1.5 g/dl where 79% (34/43) of those experienced relapse. Moreover, from those children having high triglycerides (*n* = 36), 80.6% (29/36) developed relapse. Sixty-six (37.9%) participants had hematuria in which 33.3% (22/66) of them found to be relapsed. The mean serum protein level was 4.77 g/dl ±0.96 SD, and the median serum albumin level was found to be 2 g/dl (Table 3).

### 3.4. Comparison of Survival Status Using Kaplan-Meier

**Figure 2:**
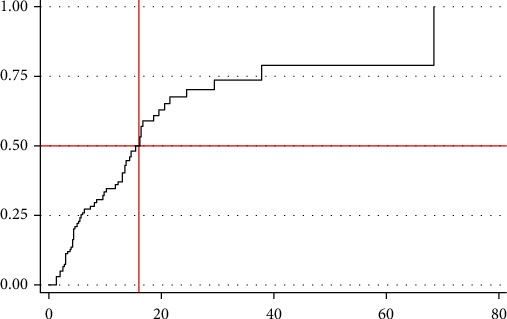
Overall Kaplan-Meier relapse estimate among children with nephrotic syndrome in comprehensive specialized hospitals of Tigray region, Northern Ethiopia, 2019 (*N* = 174). The *y*-axis represents the probability of relapse whereas the *x*-axis indicates the analysis time in months.

We can note from the graph that at the initial time of diagnosis the probability to develop relapse was lower, but as follow-up time prolonged the probability of relapse also increased. Generally, the Kaplan-Meier relapse curve increases stepwise, and it crosses the relapse function at a relapse probability of 0.5 (Figure 2).

### 3.5. Survival Function and an Incidence Density Rate of Relapse

The median follow-up period after the initial diagnosis of nephrotic syndrome was four months. The finding also revealed that 62 (35.6%) children included in the study developed relapse during the follow-up period, and the rest 112 (64.4%) were censored. Among those considered as censored (*n* = 112), 64 (36.8%) did not develop relapse up to the end of the study, 20 (11.5%) lost, 16 (9.2%) died, and 12 (6.9%) were transferred to other institutions. The total person-time observation was 1454 child-months. The incidence rate of relapse was 42.60 (95% CI 30 to 50) per 1000 child-months of observation. The median relapse time was found to be 16 months (95% CI 13-19). The cumulative probabilities of relapse at the end of 12, 24, and 36 months were 0.37, 0.69, and 0.73, respectively.

### 3.6. Predictors of Relapse

In the bivariable analysis, ten variables were included, of which hypertension and hematuria had *P* value > 0.25 and excluded from multivariate analysis. In the final Cox proportional hazard model, having undernutrition, high triglyceride level at initiation, baseline low serum albumin level ≤1.5 g/dl, and residing in rural areas were found to be independent predictors of relapse in children with nephrotic syndrome at 95% confidence level (Table 4).

Children who had undernutrition were observed to have a 3.44 times higher hazard of developing relapse for nephrotic syndrome [AHR = 3.44; 95% CI: 1.78-6.65] than their counterparts. In addition to this, children with high TG (>130 mg/dL) at the time of diagnosis had 3.37 times greater hazard than those who had borderline TG levels [AHR = 3.37; 95% CI: 1.04-10.90].

Moreover, those children who had low albumin levels (≤1.5 g/dl) at baseline were also 3.5 times at higher hazard of experiencing relapse as compared to those having serum albumin levels of >1.5 g/dl [AHR = 3.5; 95% CI: 1.81-6.80]. The other factor that showed significant association was the place of residence. Children who had been residing in rural areas were four times at an increased hazard of relapse than those who were living in urban residence [AHR = 4; 95% CI: 1.49-10.76] (Table 4).

## 4. Discussion

The incidence rate of relapse was 42.6 per 1000 child-months of observation. The median time to relapse was found to be 16 months (95% CI 13-19). Having undernutrition, high triglyceride at initiation, baseline low serum albumin level, and rural residence were found to be independent predictors of relapse.

The incidence rate of relapse was 42.6 per 1000 child-month-observations. Around 36% (95 CI: 28.80-43.09) of children with nephrotic syndrome developed relapse. This was lower than the proportion of relapse in a combined study in Europeans, South Asians, and East/Southeast Asians [29] 85%, 79%, and 70%, respectively. The possible explanation for this might be due to the difference in the length of the follow-up period, ethnicity [29], and the large area covered in the previous studies. It was also lower than the study done in Australia (80%); Indian studies conducted in 2013 (59.3%), 2014 (63%), and 2017 (88%); country of Gironde, France (83%); population-based cohort in France (79%); and Nigeria (64%) [12, 30–35]. The discrepancy with Australia and Indian studies might be due to the difference in age of the study participants, since those studies exclude children of age above 15 years unlike the current study, which incorporated up to 18 years of age; though, those children older than 15 years did not have a relapse in our cohorts during the study period. This inclusion with no events recorded might decrease the proportion of relapse in our observations.

In this study, the median time to relapse was 16 months which was in line with the study done in Nigeria which is 11 months [35]. On the contrary, it was higher than the study done in India, Australia, and France 5.5, 5, and 8.3 months, respectively [12, 30, 34]. This discrepancy might be due to the dominance of infrequent relapse cases in our study and the possible difference in identification of relapses in different settings. Furthermore, this might also be because of the difference in the length of the follow-up period. In the current study, the median follow-up time was 4 months. In this case, some patients might not be followed-up to the desired time to declare relapse and might exit before the expected time of relapse. This, in turn, could lead to the decrement of subjects that were followed for a longer period to be included in the estimation of median relapse time. Finally, the chance of relapse would increase with increased follow-up time and thus increased median relapse time.

Children who had undernutrition were observed to have a 3.44 times higher hazard of developing relapse for nephrotic syndrome [AHR = 3.44; 95% CI: 1.78-6.65] than those with no undernutrition. This finding complied with the study done in Indonesia [36]. Here, the triggering event that produces proteinuria remains unknown. However, shreds of evidence suggest its linkage to immune pathogenesis (particularly T lymphocytes), podocyte biology (glomerular filtration barrier), and genetics. Proteinuria and poor nutrition have synergistic effects. Loss of protein could result in malnutrition. On the other hand, once children are diagnosed with nephrotic syndrome, they might develop hypoproteinemia and micronutrient deficiency that leads to chronic hypoalbuminemia. Again, this decreased serum albumin, and micronutrients could lead to edema and ineffective immune response, resulting in the reoccurrence of proteinuria. In the current study finding, more than 93% of children who developed relapse had undernutrition and serum albumin levels of ≤1.5 g/dl, which will strengthen the above justification.

Children who had a high level of triglyceride level at the initial time of nephrotic syndrome diagnosis were 3.37 times at increased hazard of relapse than those who had borderline triglyceride levels. This finding is in agreement with the study done in India [37]. This could be explained by the podocyte biology theory of proteinuria pathogenesis that the elevated triglyceride level may alter the glomerular filtration barrier. Furthermore, the plasma protein loss from the primary infection will result in the synthesis of lipoproteins and low albumin levels. Then, the liver starts to produce more albumins in response to the fall in serum albumin levels. At the same time, the liver also releases more cholesterol and triglycerides that will, in turn, lead to the recurrence of signs and symptoms of nephrotic syndrome. Congruently, in this study, 88% of children who had a high level of triglyceride and serum albumin level ≤1.5 g/dl developed relapse.

Children who had baseline serum albumin level ≤1.5 g/dl were 3.5 times at higher hazard of relapse compared to their counterparts. This finding was conformable with the finding of the previous study in Dhaka medical college hospital (DMCH) [38]. This might be because albumin is the main protein in the blood, which maintains oncotic pressure, and thus, prevents leakage of fluid to the extracellular space and subsequent formation of edema. Therefore, low serum albumin level results in fluid shifting, recurrence of edema, and thus relapse [39]. In agreement with this idea, 79% of the participant who had a serum albumin level ≤1.5 g/dl in the current study developed relapse.

Children who had been residing in rural areas were four times at an increased hazard of relapse than their counterparts [AHR = 4; 95% CI 1.49-10.76]. This finding is supported by the study done on DMCH [38]. The possible explanation for this might be due to the delay in initiation of treatment as well as lack of awareness on the intake of balanced and healthy foods.

Initial steroid therapy and family history of renal disease were reported as predictors of relapse in previous studies [8, 40]. The current study did not include patients with a recognized family history of renal diseases. Likewise, all children with nephrotic syndrome incorporated in this study received prednisolone therapy in the initial attack and thus could not be analyzed in both cases. The main limitation of this study could be the recorded shorter median follow-up time that could decrease the relapse rate.

## 5. Conclusion

Relapse is a demanding situation in children with nephrotic syndrome. During the follow-up period, the overall median time to first relapse remains high. The incidence of relapse was higher in the first year of the follow-up period. Moreover, children who had undernutrition, elevated triglyceride, low serum albumin level (≤1.5 g/dl), and residing in rural areas were at a higher hazard of relapse.

## Figures and Tables

**Table 1:** Distribution of sociodemographic characteristics among children with nephrotic syndrome in comprehensive specialized hospitals of Tigray region, Northern Ethiopia, 2019 (*N* = 174).

Variables	Category	Total (%)	Status (outcome)	
			Event (%)	Censored (%)
Age at diagnosis (years)	1 to 3	23 (13.2)	9 (39.1)	14 (60.9)
	4 to 6	36 (20.7)	8 (22.2)	28 (77.8)
	7 to 9	56 (32.2)	24 (42.9)	32 (57)
	Greater than 9	59 (33.9)	21 (35.6)	38 (64.4)

Gender	Male	98 (56.3)	59 (60)	39 (40)
	Female	76 (43.7)	3 (4)	73 (96)

Residence	Urban	51 (29.3)	7 (13.7)	44 (86.3)
	Rural	123 (70.7)	55 (44.7)	68 (55.3)

**Table 2:** Disease-related characteristics of children with nephrotic syndrome in comprehensive specialized hospitals of Tigray region, Northern Ethiopia, 2019 (*N* = 174).

Variables	Category	Total (%)	Event (%)	Censored (%)
HTN	Yes	90 (51.7)	32 (35.6)	58 (64.4)
	No	84 (48.3)	30 (35.7)	54 (64.3)

Infection	Yes	102 (58.6)	58 (57)	44 (43)
	No	72 (41.4%)	4 (5.6)	68 (94.4)

History of allergy	Yes	29 (16.7%)	20 (69)	9 (31)
	No	145 (83.3%)	42 (29)	103 (71)

Time to achieve remission (days)	0-7	19 (10.9%)	4 (21)	15 (79)
	8-14	80 (46%)	21 (26.3)	59 (73.8)
	>14	75 (43.1%)	37 (49.3)	38 (50.7)

Duration of treatment (weeks)	<8	39 (22.4%)	16 (41)	23 (59)
	≥8	135 (77.6%)	46 (34)	89 (66)

Nutrition	Normal	128 (73.6)	30 (23.4)	98 (76.6)
	Undernutrition	46 (26.4)	32 (69.6)	14 (30.4)

Abbreviation: HTN: hypertension.

**Table 3:** Distribution of biochemical-related characteristics among children with nephrotic syndrome in comprehensive specialized hospitals of Tigray region, Northern Ethiopia, 2019 (*N* = 174).

Variables	Category	Total (%)	Event (%)	Censored (%)
Protein (g/dL)	<3	8 (4.6)	5 (62.5)	3 (37.5)
	3-6	158 (90.8)	55 (34.8)	103 (65.2)
	>6	8 (4.6)	2 (25)	6 (75)

Albumin (g/dL)	≤1.5	43 (24.7)	34 (79)	9 (21)
	>1.5	131 (75.3)	28 (21.4)	103 (78.6)

TGD (mg/dL)	Normal (<110)	43 (24.7)	5 (11.6)	38 (90.5)
	Borderline (110-129)	95 (55)	28 (29.47)	67 (69.8)
	High (≥130)	36 (20.7)	29 (80.6)	7 (19.4)

Hematuria	Yes	66 (37.9)	22 (33.3)	44 (66.7)
	No	108 (62.1)	40 (37)	68 (63)

Abbreviation: g/dl: grams per deciliter; TGD:triglyceride.

**Table 4:** Bivariable and multivariate analysis output for children with nephrotic syndrome in comprehensive specialized hospitals of Tigray region, Northern Ethiopia, 2019 (*N* = 174).

		Outcome status		Crude HR (95% CI)	Adjusted HR (95% CI)
Covariates	Category	Relapsed	Censored		

Age (in years)	1-3	9	14	1.77 (0.78-4.00)	2.24 (0.86-5.83)
	4-6	8	28	0.84 (0.36- 1.93)	2.65 (0.99-7.11)
	7-9	24	32	1.52 (0.83-2.79)	1.33 (0.66-2.68)
	>9	21	38	—	—

Residence	Urban	51	7	—	—
	Rural	123	55	2.9 (1.30-6.40)	4 (1.49-10.76)^∗^

History of allergy	Yes	29	20	0.39 (0.22- 0.68)	1.18 (0.61-2.28)
	No	145	42	—	—

Time to achieve remission (days)	0-7	19	4	—	—
	8-14	80	21	1.63 (0.96-2.78)	0.90 (0.27-2.99)
	>14	75	37	0.74 (0.37-1.48)	1.18 (0.37-3.76)

Duration of treatment	<8 weeks	39	16	0.65 (0.36-1.18)	0.74 (0 .37-1.50)
	≥8 weeks	135	46	—	—

Albumin (g/dL)	≤1.5	43	34	4.14 (2.44-7.00)	3.51 (1.81-6.80)∗
	>1.5	131	28	—	—

TGD	Normal	43	5	—	—
	Borderline	95	28	4.6 (1.38-15.28)	1.66 (0.53-5.2)
	High	36	29	9.78 (2.94-32.55)	3.37 (1.04-10.9)^∗^

Nutrition	Normal	128	30	—	—
	Malnourished	46	32	3.39 (2.01-5.71)	3.44 (1.78-6.65)^∗^

Note: ^∗^significant at 5% level of significance. Abbreviations: HR: hazard ratio; CI: confidence interval; g/dl: grams per deciliter; TGD: triglyceride.

## Data Availability

Extra data that support the findings of this study are available from the corresponding author upon reasonable request and can be shared upon legal request via milieteg3@gmail.com.

## References

[1] Muoneke V., Una A. F., Eke C. B., Anyanwu O. U. (2016). The burden and outcome of pediatric renal admissions at the federal teaching hospital Abakaliki: a 3-year review (2011–2013). *Annals of Medical and Health Sciences Research*.

[2] van Biljon G. (2011). Nephrotic syndrome in children–studies from South Africa. *An Update on Glomerulopathies-Clinical and Treatment Aspects*.

[3] Bofarraj M. A. M., Khaial F. S. B., Abduljawad N. H., Alshowbki R. (2018). Childhood nephrotic syndrome -a single centre experience in Althawra central hospital, AlbaidaLibya during 2005-2016. *MOJ Surgery*.

[4] Moorani K. N. (2011). Infections are common as a cause of relapse in children with Nephrotic syndrome. *Pakistan Pediatric Journal*.

[5] Eddy A. A., Symons J. M. (2003). Nephrotic syndrome in childhood. *The Lancet*.

[6] Kidney Disease (2012). *Improving Global Outcomes (KDIGO) Clinical Practice Guideline for Glomerulonephritis*.

[7] Rajendra N. P., Anant G. B. (2017). A study of clinical profile and associated factors of nephrotic syndrome in children at the tertiary health care center. *MedPulse International Journal of Pediatrics*.

[8] Fomina S., Pavlenko T., Englund E., Bagdasarova I. (2011). Clinical course of steroid-sensitive nephrotic syndrome in children: outcome and outlook. *The Open Pediatric Medicine Journal*.

[9] Pais P., Avner E. D., Kliegman R. M., Stanton B. F., St. Geme J., Schor N. F. (2016). Nelson Textbook of Pediatrics. *Nephrotic syndrome. First South Asia Edition*.

[10] Esezobor C., Ladapo T., Lesi F. (2016). Frequency of relapse among Nigerian children with steroid-sensitive nephrotic syndrome. *Nigerian Journal of Clinical Practice*.

[11] Pasini A., Benetti E., Conti G. (2017). The Italian Society for Pediatric Nephrology (SINePe) consensus document on the management of nephrotic syndrome in children: part I-diagnosis and treatment of the first episode and the first relapse. *Italian Journal of Pediatrics*.

[12] Sureshkumar P., Hodson E. M., Willis N. S., Barzi F., Craig J. C. (2014). Predictors of remission and relapse in idiopathic nephrotic syndrome: a prospective cohort study. *Pediatric Nephrology*.

[13] Uwaezuoke S. N., Okafor H. U., Eneh C. I., Odetunde O. I. (2016). The triggers and patterns of relapse in childhood idiopathic nephrotic syndrome. *Journal of Clinical Nephrology*.

[14] Balaji J., Kumaravel K. S., Punitha P., Rameshbabu B. (2017). Risk factors for relapse in childhood steroid-sensitive nephrotic syndrome. *Indian Journal of Child Health*.

[15] Ali E. M. A., Nahla M. E., Mohamed B. A., Rashid A. E. (2018). Childhood seroidsensitive nephrotic syndrome: characteristics and predictors of relapses a study at a single center in Khartoum Sudan. *Journal of Medical Sciences*.

[16] Ishikura K., for Japanese Study Group of Renal Disease in Children, Yoshikawa N. (2015). Morbidity in children with frequently relapsing nephrosis: 10-year follow-up of a randomized controlled trial. *Pediatric Nephrology*.

[17] Kyrieleis H. A. C., Löwik M. M., Pronk I. (2009). Long-term outcome of biopsy-proven, frequently relapsing minimal-change nephrotic syndrome in children. *Clinical Journal of the American Society of Nephrology*.

[18] Saha A., Gupta V., Kapoor K. (2013). Hearing status in children with frequently relapsing and steroid resistant nephrotic syndrome. *Pediatric Nephrology*.

[19] Agrawal S., Krishnamurthy S., Naik B. N. (2017). Assessment of quality of life in children with nephrotic syndrome at a teaching hospital in South India. *Saudi Journal of Kidney Diseases and Transplantation*.

[20] Mitra S., Banerjee S. (2011). The impact of pediatric nephrotic syndrome on families. *Pediatric Nephrology*.

[21] Mishra K., Ramachandran S., Firdaus S., Rath B. (2015). The impact of pediatric nephrotic syndrome on parents’ health-related quality of life and family functioning: an assessment made by the PedsQL 4.0 family impact module. *Saudi Journal of Kidney Diseases and Transplantation*.

[22] Sherali A. R., Moorani K. N., Chishty S. H., Khan S. I. (2014). Zinc supplement in the reduction of relapses in children with steroid-sensitive nephrotic syndrome. *Journal of the College of Physicians and Surgeons–Pakistan*.

[23] Mola K., Shimelis D. (2016). Pattern and outcome of renal diseases in hospitalized children in Tikur anbessa specialized teaching hospital, Addis Ababa, Ethiopia. *Ethiopian Medical Journal*.

[24] Tigray Regional Health Bureau (2018). *Regional estimates of the population with a conversion factor. Unpublished resources*.

[25] KDIGO (2012). Improving global outcomes: clinical practice guideline for the Evaluation and Management of acute kidney injury. *Kidney International Supplements*.

[26] Nourbakhsh N., Mak R. H. (2017). Steroid-resistant nephrotic syndrome: past and current perspectives. *Pediatric Health, Medicine and Therapeutics*.

[27] Federal Ministry of health (2007). *Protocol for the management of severe acute malnutrition*.

[28] Organization WH (2008). *Training Course on Child Growth Assessment*.

[29] Banh T. H. M., Hussain-Shamsy N., Patel V. (2016). Ethnic differences in incidence and outcomes of childhood nephrotic syndrome. *Clinical Journal of the American Society of Nephrology*.

[30] Mishra O. P., Abhinay A., Mishra R. N., Prasad R., Pohl M. (2013). Can we predict relapses in children with idiopathic steroid-sensitive nephrotic syndrome?. *Journal of Tropical Pediatrics*.

[31] Sahana K. S. (2014). Clinical profile of nephrotic syndrome in children. *Journal of Evolution of Medical and Dental Sciences*.

[32] Prasun B., Payas J., Sujaya M. (2017). Prediction of relapses in children with idiopathic steroid-sensitive nephrotic syndrome: a retrospective study. *International Journal of Contemporary Pediatrics*.

[33] Ernould S., Godron A., Nelson J. R., Rigothier C., Llanas B., Harambat J. (2011). Idiopathic nephrotic syndrome in children: incidence, clinical presentation, and outcome in the county of Gironde, France. *Archives de Pédiatrie*.

[34] Dossier C., Delbet J. D., Boyer O. (2019). Five-year outcome of children with idiopathic nephrotic syndrome: the NEPHROVIR population-based cohort study. *Pediatric Nephrology*.

[35] WasiuA O., KayodeA A., Olufemi A. (2010). Reversed clinical and morphologic characteristics of idiopathic childhood nephrotic syndrome. *Nephro-Urology Monthly*.

[36] Albar H., Bilondatu F., Daud D. (2018). Risk factors for relapse in pediatric nephrotic syndrome. *Paediatrica Indonesiana*.

[37] Suresh P. M., Subasakthi A., Prasath S. V. A., Anandan H. (2017). Plasma lipid profile - prognostic factor in nephrotic syndrome – a prospective study. *Annals of International medical and Dental Research*.

[38] Sarker M., Islam M., Saad T. (2012). Risk factor for relapse in childhood nephrotic syndrome-a hospital-based retrospective study. *Faridpur Medical College Journal*.

[39] Kliegman (2015). *Nelson textbook of pediatrics*.

[40] Rahi K., Al-Badri A. A. S., Salih B. J., Hasan F. O. (2009). Childhood nephrotic syndrome, frequent and infrequent relapses, and risk factors for relapses. *Iraqi Academic Scientific Journal*.

